# Rifampin Reduces the Plasma Concentrations of Oral and Intravenous Hydromorphone in Healthy Volunteers

**DOI:** 10.1213/ANE.0000000000005229

**Published:** 2020-11-06

**Authors:** Terhi J. Lohela, Satu Poikola, Mikko Neuvonen, Mikko Niemi, Janne T. Backman, Klaus T. Olkkola, Tuomas O. Lilius

**Affiliations:** From the * Department of Clinical Pharmacology, Faculty of Medicine; † Department of Anaesthesiology, Intensive Care Medicine, and Pain Medicine, University of Helsinki and HUS Helsinki University Hospital, Helsinki, Finland; ‡ Individualized Drug Therapy Research Program, Faculty of Medicine, University of Helsinki, Helsinki, Finland.

## Abstract

Several opioids are metabolized by the inducible cytochrome P450 (CYP) 3A isozymes. Coadministration with strong inducers of drug metabolism, such as rifampin, can dramatically reduce systemic exposure to these opioids. As the CYP metabolism of hydromorphone is of minor importance, we studied in healthy volunteers whether hydromorphone would be an effective analgesic for patients who concomitantly receive the prototypical enzyme inducer rifampin.

**METHODS:**

In this paired, randomized, crossover study, 12 participants received oral placebo or rifampin for 8 days. Oral hydromorphone (2.6 mg) was administered on day 6 followed by intravenous hydromorphone (0.02 mg/kg) on day 8. Hydromorphone and hydromorphone-3-glucuronide (HM3G) plasma concentrations were measured for 24 hours and psychomotor responses, including perceived drug effect, change in pupil diameter, and cold pressor threshold were evaluated for 6 hours. Our primary outcome was the change in the area under the concentration–time curve (AUC_0–last_) of oral and intravenous hydromorphone after pretreatment with rifampin or placebo. Pharmacodynamic parameters and other pharmacokinetic parameters were analyzed as secondary outcomes.

**RESULTS:**

Rifampin reduced the AUC_0–last_ of oral and intravenous hydromorphone by 43% (ratio to control: 0.57, 90% confidence interval [CI], 0.50-0.65) and 26% (ratio to control: 0.74, 90% CI, 0.69-0.79), respectively. The maximum concentration of oral hydromorphone was reduced by 37% (ratio to control: 0.63, 90% CI, 0.55-0.72), and oral bioavailability decreased from 33% to 26% (ratio to control: 0.78, 90% CI, 0.67-0.91) in the rifampin phase compared with placebo. The HM3G-to-hydromorphone ratio increased by 50% (90% CI, 25-79) and 42% (90% CI, 29-55) after oral and intravenous hydromorphone, respectively. Rifampin did not significantly affect the pharmacodynamic parameters.

**CONCLUSIONS:**

Rifampin significantly reduces the concentrations of oral and intravenous hydromorphone. This interaction is due to an increase in the first-pass and systemic metabolism of hydromorphone, likely involving induction of uridine 5′-diphospho- glucuronosyltransferase enzymes by rifampin. The enhancement of hydromorphone elimination should be considered when managing pain of patients who are treated with strong enzyme inducers.

**KEY POINTS**

- **Question:** Does rifampin affect the pharmacokinetics of oral and intravenous hydromorphone?
- **Findings:** In healthy volunteers, rifampin reduced exposure to oral and intravenous hydromorphone by 43% and 26%, respectively.
- **Meaning:** This reduction in hydromorphone exposure should be considered when managing patients who are cotreated with strong enzyme inducers.

The majority of clinically used opioids are metabolized by the highly inducible cytochrome P450 (CYP) 3A isozymes in the liver and the small intestine.^1–5^ Coadministration with strong inducers of drug metabolism, such as rifampin, can dramatically reduce the systemic exposure to these opioids, including buprenorphine, fentanyl, methadone, oxycodone, and tramadol, possibly leading to poor analgesic efficacy.^6–12^

Hydromorphone, a strong μ-opioid receptor agonist, is mainly used for postoperative and cancer pain. It is 5–7 times more potent than morphine^13–15^ and is metabolized mainly by glucuronidation to hydromorphone-3-glucuronide (HM3G) by uridine 5′-diphospho-glucuronosyltransferase 2B7 (UGT2B7).^16^ HM3G has neuroexcitatory properties and has been proposed to play a role in the development of analgesic tolerance and hyperalgesia to hydromorphone.^17,18^ After oral administration, the bioavailability of hydromorphone is highly variable, ranging from 13% to 62%.^19^ Because the CYP metabolism of hydromorphone is minor, the reduction in the hydromorphone exposure by rifampin should theoretically be small. However, rifampin has been shown to decrease systemic exposure to morphine, another opioid with glucuronidation as a major pathway of elimination,^20^ implicating that other mechanisms may be involved behind the effects of rifampin. Nevertheless, in the absence of previous evidence, some pain centers have recommended hydromorphone as a primary strong opioid analgesic for patients who are treated with enzyme inducers.

The aim of the study was to assess in healthy volunteers, whether rifampin affects the pharmacokinetics of hydromorphone. As the CYP metabolism of hydromorphone is considered minor, our hypothesis was that rifampin does not significantly influence hydromorphone concentrations or its effects.

## METHODS

### Ethics

This trial was conducted according to the revised Declaration of Helsinki. It was approved by the Coordinating Ethics Committee of the Helsinki and Uusimaa Hospital District (record number HUS/332/2017) and the Finnish Medicines Agency Fimea. Written informed consent was obtained from all subjects participating in the trial. The trial was registered before volunteer enrollment at the EudraCT database (Supplemental Digital Content 1, Document, http://links.lww.com/AA/D207, EudraCT number 2016-004841-97, principal investigator: Klaus T. Olkkola, date of registration: January 20, 2017). The study was performed from March 2017 to May 2017 in the facilities of the Department of Clinical Pharmacology, Helsinki University Hospital, Helsinki, Finland, and Department of Anaesthesiology, Intensive Care Medicine, and Pain Medicine, Helsinki University Hospital, Helsinki, Finland. We adhered to the applicable Consolidated Standards of Reporting Trials (CONSORT) guidelines.

### Subjects

Twelve healthy volunteers (5 women and 7 men; age range: 20–34 years; weight range: 56–100 kg) were determined to be in good health by medical history, clinical examination, electrocardiography, and routine laboratory screens. Urine toxicology and pregnancy test results were negative. None of the participants was taking any regular medication or a smoker. The risk of participants to develop opioid abuse was low as assessed by the Finnish translation of the Abuse Questions.^21^

### Study Design

We conducted a 2-phase, paired, double-blinded, crossover study (Supplemental Digital Content 2, Figure 1, http://links.lww.com/AA/D257) with an interval of 6 weeks between the sessions. The participants were given in randomized order either 600 mg oral rifampin (Rimapen; Orion, Espoo, Finland) or placebo (Placebo tablets; University Pharmacy, Helsinki, Finland) once daily at 8 pm for 8 days. The volunteers and investigators were blinded to the placebo, and rifampin pretreatments and drug concentration analyses were performed blinded. The data were analyzed unblinded.

During each of the 2 phases (ie, rifampin and placebo phase), hydromorphone was administered twice: orally on day 6 and intravenously on day 8. On day 6 of each phase, participants received 2.6 mg oral hydromorphone hydrochloride (Palladon, 2.6 mg capsules; Mundipharma, Vantaa, Finland) at 8 am, 12 hours after the fifth dose of rifampin or placebo. Two more doses of rifampin or placebo were given on day 6 and 7. On day 8 at 8 am, the participants were administered 0.02 mg·kg^–1^ intravenous hydromorphone hydrochloride (Palladon, 2 mg/mL; Mundipharma) as a slow injection followed by a saline flush. The median intravenous dose of hydromorphone was 1.5 mg (interquartile range: 1.3–1.9 mg). Intravenous hydromorphone was administered in a recovery room of an operating theatre, and pulse oximetry, blood pressure, and heart rate were monitored for 4 hours following hydromorphone.

To confirm the induction of CYP enzymes, participants also received a small dose of midazolam (Midazolam Accord, 1 mg/mL, Accord, Middlesex, UK), a typical probe for CYP3A enzyme induction,^22^ both on days 6 and 8. A dose of 0.1 mg of oral midazolam diluted to 150 mL of lukewarm water was administered at the same time with hydromorphone.

Adherence with the rifampin or placebo dosing schedule was ascertained by the use of text messages. The participants fasted for 8 hours before the administration of hydromorphone. They were given standard meals 4 and 8 hours after hydromorphone. Smoking, use of drugs or alcohol, or the consumption of grapefruit juice was prohibited during the study.

### Blood Sampling

On the test days, timed blood samples were drawn from a cannulated forearm vein into Ethylenediaminetetraacetic acid (EDTA) tubes immediately before and 0.5, 1, 1.5, 2, 3, 4, 5, 6, 8, 10, 12, and 24 hours after administration of hydromorphone and midazolam. An additional blood sample was drawn 0.25 hours after intravenous hydromorphone administration. For intravenous hydromorphone administration, a venous cannula was inserted into the hand opposite to the arm used for drug sampling. Tubes were placed immediately on ice, and plasma was separated within 30 minutes and stored at −70°C until analysis.

### Determination of Drug Concentrations

Authentic reference standards, hydromorphone, HM3G, and midazolam, and the corresponding isotope-labeled internal standards hydromorphone-13C-D3, HM3G-D5, and midazolam-D6 were purchased from Toronto Research Chemicals (North York, ON, Canada). The reference standard samples and quality control samples were prepared in plasma and pretreated in a similar way to the subject plasma samples. Before quantification, a volume of 0.2 mL plasma sample was mixed with 0.2 mL of internal standard solution (2 ng/mL each in 0.1 N HCl), and the sample was extracted using Oasis MCX µElution 96-well plate according to the manufacturer’s instructions (Waters Corp, Milford, MA). Briefly, the solid phase extraction plate was conditioned with methanol and water, and samples were loaded into the plate. The wells were then washed with 0.1 N HCl (200 µL) and 100% methanol (200 µL), and the analytes were eluted into a 96-well collection plate with 2 × 30 µL of 5% ammonium hydroxide in methanol. The sample extracts were evaporated to dryness using a centrifugal evaporator (GeneVac, Thermo Fisher Scientific, Waltham, MA) and reconstituted in 30 µL of 20% methanol.

The drug concentrations were measured using a Nexera X2 UHPLC system (Shimadzu, Kyoto, Japan) coupled to a 5500 Qtrap mass spectrometer interfaced with an electrospray ion source (ABSciex, Toronto, ON). The analytes were separated on an XBridge C18 column (3.5 µm particle size, 2.1 × 100 mm internal diameter; Waters Corp, Milford, MA) using gradient elution. The mobile phase consisted of 5 mM ammonium formate (pH 8.8) and acetonitrile, and the mobile phase gradient was set as follows: 10% acetonitrile on hold over 2 minutes, followed by a linear ramp of acetonitrile from 10% to 90% over 6 minutes and re-equilibration back to the starting composition.

The flow rate and the column temperature were maintained at 300 µL/min and 30°C, and an aliquot of 8 µL was injected into the system. The mass spectrometer was operated in a positive polarity mode and the targeted mass-to-charge (m/z) ion transitions were 286–185 for hydromorphone, 462–286 for HM3G, and 326–291 for midazolam. The limits of quantification (LOQs) were 0.05, 0.1, and 0.005 ng/mL, respectively. The assay was linear (*r* > 0.998) over the measured concentration range for all analytes, and the day-to-day (n = 6) coefficients of variation (CV%) were 7.1% (0.2 ng/mL) and 5.0% (2.0 ng/mL) for hydromorphone, 11.3% (0.2 ng/mL) and 6.0% (10 ng/mL) for HM3G, and 8.6% (0.02 ng/mL) and 5.4% (0.2 ng/mL) for midazolam.

### Pharmacokinetic Analysis

The maximum plasma concentration (*C*_max_), the time to reach maximum concentration (*t*_max_), area under the concentration–time curve from start to the last detectable concentration of hydromorphone (AUC_0–last_), and elimination half-life (*t*_½_) were calculated for hydromorphone, HM3G, and midazolam by standard noncompartmental methods using Phoenix WinNonlin, v. 6.4 (Certara, Princeton, NJ). The AUC_0–last_ was calculated using the linear-up log-down trapezoidal method, and *t*_½_ was calculated from the log-linear part of the elimination curve. The oral bioavailability (*F*) of hydromorphone was calculated using the following formula: *F* = (AUC_0–last oral_ · Dose_intravenous_)/(AUC_0–last intravenous_ · Dose_oral_). After oral administration of hydromorphone, the apparent oral clearance (Cl/*F*) was calculated using the following formula: Cl/*F* = Dose_oral_/AUC_0–last oral_. The apparent volume of distribution of hydromorphone during elimination (*V*_z_/*F*) was calculated using the following formula: *V*_z_/*F* = Dose_oral_/(AUC_0–last oral_ · *k*_el_), where *k*_el_ is the elimination constant.

### Pharmacodynamic Measurements

As a secondary outcome, 3 pharmacodynamic parameters, perceived drug effect (assessed with a 100-mm visual analog scale), pupil diameter, and cold pressor threshold were assessed at baseline and 0.5, 1, 1.5, 2, 3, 4, 5, and 6 hours after oral and intravenous hydromorphone.

Pupil diameter was measured using a pupillometer (NeurOptics PLR-200, Irvine, CA) under a controlled ambient light environment. The measurement was repeated twice per time point, and the higher measured diameter value was used. During the measurements, the contralateral eye was covered with an eye patch.

Cold pressor threshold was determined to evaluate cold pain sensitivity. In the test, the volunteer immersed their dominant hand into ice water (temperature ranging from 0°C to 2°C) up to the wrist for 1 minute. The delay in seconds to the first painful sensation was recorded as the cold pain threshold. The areas under effect–time curve (AUC_0–6_) for all 3 pharmacodynamic parameters were determined by the trapezoidal rule for 0–6 hours. Some volunteers reported lower pain thresholds after drug administration compared with baseline, and, as a result, the reported AUC_0–6_ values are negative for these individuals.

### Statistical Analysis

The data were analyzed as between-phase comparisons for each individual. In line with the standard practice for reporting pharmacokinetic comparisons,^23^ pharmacokinetic results are expressed as geometric means with geometric CV or geometric mean ratios with 90% confidence intervals (90% CI), except *t*_max_ for which median and range are. The geometric mean ratio and its 90% CI were obtained through first calculating the difference between the logarithms of the geometric means and a 90% CI for this difference. The antilogarithms of the difference and the 90% CI were then calculated to obtain the geometric mean ratio and the respective 90% CI.

The pharmacodynamic data are presented as median AUC_0–6_ with ranges. In all analyses, we report 2-sided *P* values at 5% level. Based on the results of previous pharmacokinetic drug–drug interaction studies,^10^ 12 subjects were estimated to be adequate to detect a 30% average change in the area under the curve (AUC) of hydromorphone between the rifampin and placebo phases, with a power of at least 80% (at 5% level).

The pharmacodynamic parameters and *t*_max_ of placebo and rifampin phases were compared using Wilcoxon matched-pairs signed-ranks tests. All other pharmacokinetic parameters were compared using paired *t* tests on log-transformed variables. Statistical analyses were conducted using Stata version 15.1/MP2 (StataCorp, College Station, TX). Graphs were drawn using GraphPad Prism version 7.0e (GraphPad Software, La Jolla, CA).

## RESULTS

### Effect of Rifampin on Pharmacokinetics of Oral Hydromorphone

**Figure 1.**
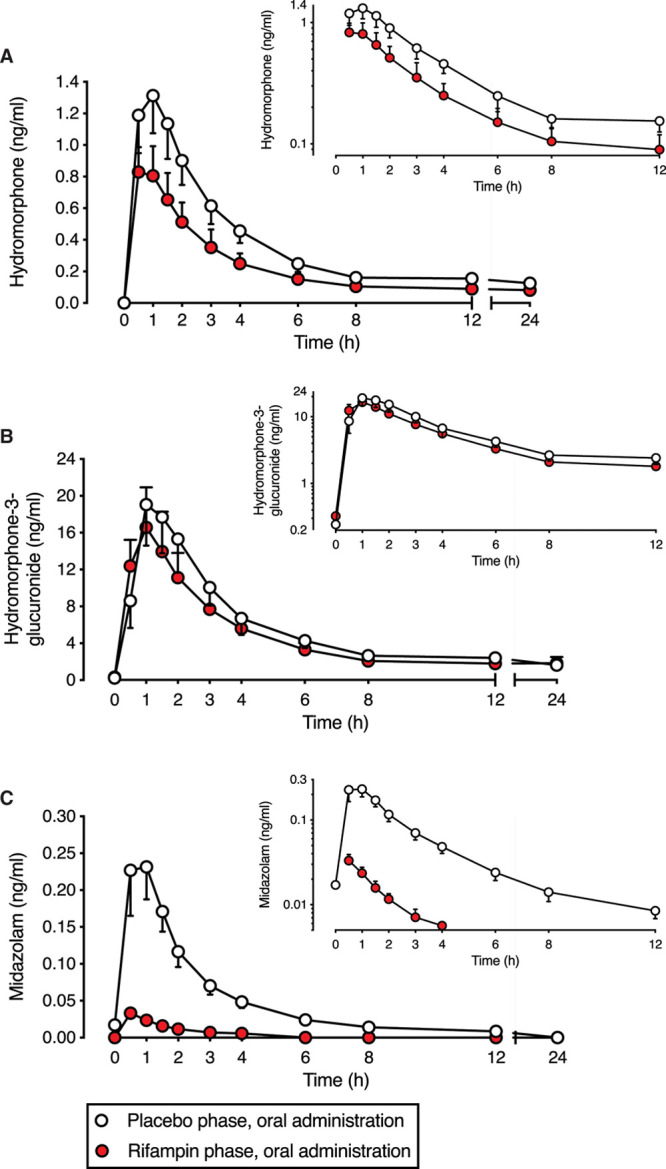
Rifampin reduces the plasma concentrations of oral hydromorphone. Effect of rifampin pretreatment on plasma concentrations of (A) hydromorphone, (B) hydromorphone-3-glucuronide, and (C) midazolam after oral hydromorphone (2.6 mg) and oral midazolam (0.1 mg) in 12 healthy participants. Data are presented as geometric means with 90% confidence intervals. Insets show the same data on a semilogarithmic scale.

All volunteers completed the study. Rifampin reduced the concentrations of oral hydromorphone and increased the formation of HM3G compared with placebo (Figure 1). The AUC_0–last_ of oral hydromorphone fell from 6.6 ng·h·mL^–1^ in the placebo phase to 3.8 ng·h·mL^–1^ in the rifampin phase, corresponding to a decrease of 43% (ratio to control: 0.57, 90% CI, 0.50-0.65) (Table 1). Rifampin reduced the *C*_max_ of oral hydromorphone by 37% (ratio to control: 0.63, 90% CI, 0.55-0.72) and the mean *F* by 22% (ratio to control: 0.78, 90% CI, 0.67-0.91), relative to the placebo phase values. Hydromorphone *t*_max_ was 1 hour (range 0.5–1.5) in the placebo phase and 0.5 hours (range 0.5–1.5) in the rifampin phase; however, this difference was not statistically significant. The reduction in individual hydromorphone AUC_0–last_ of each participant is shown in Supplemental Digital Content 2, Figure 2A, http://links.lww.com/AA/D257.

**Table 1.** Pharmacokinetic Parameters of Hydromorphone, HM3G, and Midazolam After Oral Administration of Hydromorphone Following Placebo (Control) or Oral Rifampin in Healthy Volunteers

Parameters	Placebo Phase (Control)	Rifampin Phase	*P*
Hydromorphone			
*C*_max_ (ng/mL)	1.48 (37)	0.93 (35)	
Ratio to control (90% CI)	1	0.63 (0.55-0.72)	*<*.0001
*t*_max_ (h)	1.0 (0.5-1.5)	0.50 (0.5-1.5)	.1757
*t*_½_ (h)	3.06 (22)	2.85 (36)	
Ratio to control (90% CI)	1	0.93 (0.83-1.05)	.2987
AUC_0–last_ (ng·h·mL^–1^)	6.60 (39)	3.77 (46)	
Ratio to control (90% CI)	1	0.57 (0.50-0.65)	<.0001
Cl/*F* (L/min)	5.78 (38)	10.07 (46)	
Ratio to control (90% CI)	1	1.74 (1.53-1.99)	<.0001
*V*_z_/*F* (L)	1528 (35)	2486 (38)	
Ratio to control (90% CI)	1	1.63 (1.38-1.92)	.0003
*F*	0.33 (21)	0.26 (28)	
Ratio to control (90% CI)	1	0.78 (0.67-0.91)	.0171
HM3G			
*C*_max_ (ng/mL)	20.99 (42)	17.49 (45)	
Ratio to control (90% CI)	1	0.83 (0.71-0.97)	.0579
*t*_max_ (h)	1.25 (1.0-2.0)	1.00 (0.5-2.0)	.2235
*t*_½_ (h)	3.73 (26)	3.60 (38)	
Ratio to control (90% CI)	1	0.96 (0.81-1.15)	.7091
AUC_0–last_ (ng·h·mL^–1^)	102.08 (40)	87.28 (39)	
Ratio to control (90% CI)	1	0.86 (0.77-0.95)	.0200
HM3G-to-hydromorphone metabolic ratio (AUC_m_/AUC_p_)	15.47 (30)	23.12 (24)	
Ratio to control (90% CI)	1	1.50 (1.25-1.79)	.0021
Midazolam			
C_max_ (ng/mL)	0.28 (46)	0.03 (33)	
Ratio to control (90% CI)	1	0.12 (0.10-0.15)	<.0001
*t*_max_ (h)	1.0 (0.5-1.0)	0.50 (0.5-0.5)	.0082
*t*_½_ (h)	2.13 (19)	1.03 (23)	
Ratio to control (90% CI)	1	0.48 (0.41-0.56)	<.0001
AUC_0–last_ (ng·h·mL^–1^)	0.65 (38)	0.05 (32)	
Ratio to control (90% CI)	1	0.07 (0.06-0.09)	<.0001

Data are presented as geometric means with geometric coefficients of variation (expressed as a percentage), except *t*_max_ for which median and range are shown. Wilcoxon matched-pairs signed-ranks tests were used to compare *t*_max_ between rifampin and placebo phases. Other pharmacokinetic variables were compared using paired *t* tests on log-transformed data. Geometric mean ratios between the 2 phases are given with 90% CI.

Abbreviations: AUC_0–last_, area under the concentration–time curve; AUC_m_/AUC_p_, ratio between metabolite AUC and parent drug AUC; *C*_max_, maximum plasma concentration; CI, confidence interval; Cl/*F*, apparent oral clearance; *F*, oral bioavailability; HM3G, hydromorphone-3-glucuronide; *t*_½_, elimination half-life; *t*_max_, time to reach maximum concentration; *V*_*z*_/*F*, apparent volume of distribution of hydromorphone during elimination.

The HM3G-to-hydromorphone metabolic ratio was 50% higher (ratio to control: 1.50, 90% CI, 1.25-1.79) in the rifampin group compared with placebo. Rifampin reduced the AUC_0–last_ of HM3G by 14% (ratio to control: 0.86, 90% CI, 0.77-0.95) compared with placebo. The *C*_max_ of HM3G was 17% lower (ratio to control: 0.83, 90% CI, 0.71-0.97) in the rifampin phase compared with placebo, but this did not reach statistical significance (*P* = .0579). The *t*_max_ and *t*_½_ of HM3G were not influenced by rifampin.

Rifampin pretreatment caused clear reductions in midazolam exposure. Compared with placebo, the *C*_max_ fell by 88% (ratio to control: 0.12, 90% CI, 0.10-0.15), AUC_0–last_ by 93% (ratio to control: 0.07, 90% CI, 0.06-0.09), and t_½_ by 52% (ratio to control: 0.48, 90% CI, 0.41-0.56) in the rifampin phase.

### Effect of Rifampin on Pharmacokinetics of Intravenous Hydromorphone

**Figure 2.**
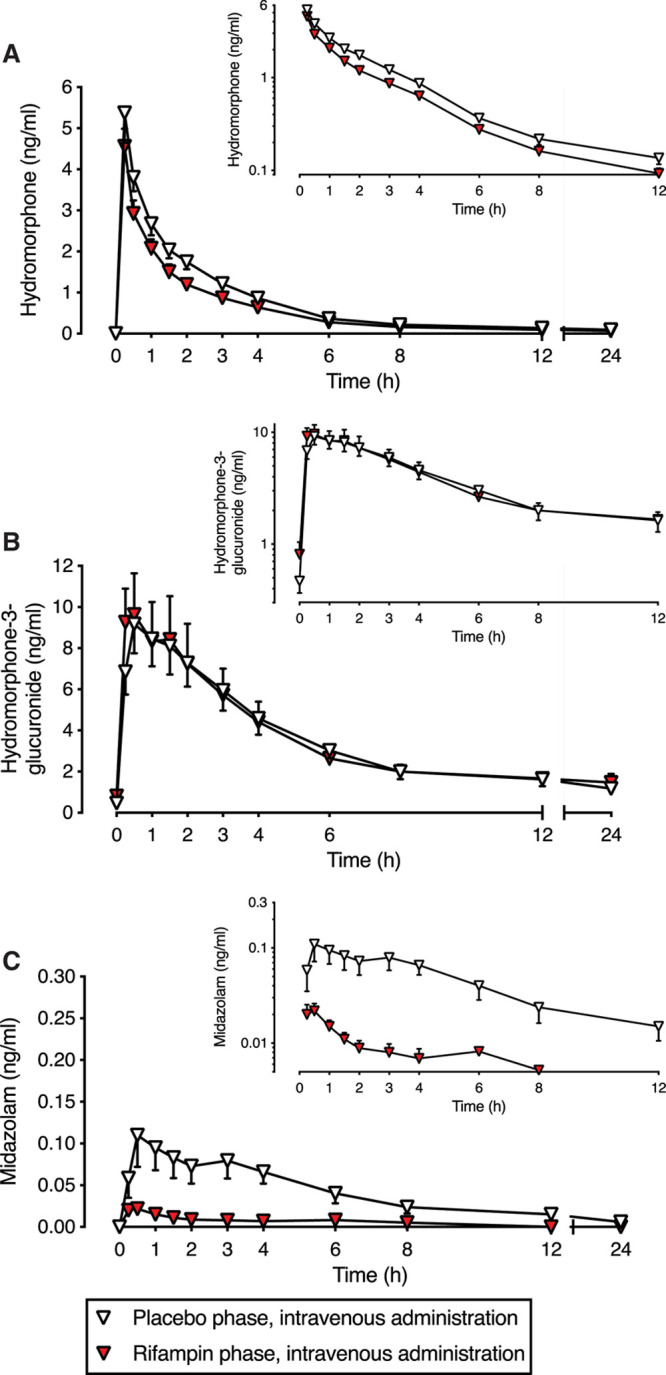
Rifampin reduces the plasma concentrations of intravenous hydromorphone. Effect of rifampin pretreatment on plasma concentrations of (A) hydromorphone, (B) hydromorphone-3-glucuronide, and (C) midazolam after intravenous hydromorphone (0.02 mg/kg) and oral midazolam (0.1 mg) in 12 healthy volunteers. Data are presented as geometric means with 90% confidence intervals. Insets show the same data on a semilogarithmic scale.

Compared with placebo, rifampin increased the clearance of intravenous hydromorphone by 36% (ratio to control: 1.36, 90% CI, 1.26-1.45), steady-state volume of distribution by 28% (ratio to control: 1.28, 90% CI, 1.14-1.44), and HM3G-to-hydromorphone metabolic ratio by 42% (ratio to control: 1.42, 90% CI, 1.29-1.55) (Figure 2; Table 2). Rifampin reduced the AUC_0–last_ of intravenous hydromorphone by 26% (ratio to control: 0.74, 90% CI, 0.69-0.79). The *t*_½_ of hydromorphone and HM3G were similar in the rifampin and placebo phases. There were no differences in the *C*_max_, *t*_max_, or AUC_0–last_ of HM3G in the rifampin phase compared with placebo.

**Table 2.** Pharmacokinetic Parameters of Hydromorphone, HM3G, and Midazolam After Intravenous Administration of Hydromorphone Following Placebo (Control) or Oral Rifampin in Healthy Volunteers

Parameters	Placebo Phase (Control)	Rifampin Phase	*P*
Hydromorphone			
Cl (L/min)	1.88 (26)	2.55 (25)	
Ratio to control (90% CI)	1	1.36 (1.26-1.45)	<.0001
*V*_ss_ (L)	492 (30)	631 (29)	
Ratio to control (90% CI)	1	1.28 (1.14-1.44)	.0034
AUC_0–last_ (ng·h·mL^–1^)	11.85 (14)	8.72 (14)	
Ratio to control (90% CI)	1	0.74 (0.69-0.79)	<.0001
*t*_½_ (h)	2.42 (12)	2.38 (10)	
Ratio to control (90% CI)	1	0.98 (0.94-1.03)	.5425
HM3G			
C_max_ (ng/mL)	9.86 (31)	10.47 (37)	
Ratio to control (90% CI)	1	1.06 (0.94-1.20)	.3848
*t*_max_ (h)	0.75 (0.5-2.0)	0.50 (0.25-2.0)	.1559
*t*_½_ (h)	3.88 (25)	4.11 (30)	
Ratio to control (90% CI)	1	1.06 (0.94-1.19)	.4152
AUC_0–last_ (ng·h·mL^–1^)	64.49 (31)	67.04 (28)	
Ratio to control (90% CI)	1	1.04 (0.93-1.17)	.5729
HM3G-to-hydromorphone metabolic ratio (AUC_m_/AUC_p_)	5.44 (24)	7.69 (29)	
Ratio to control (90% CI)	1	1.42 (1.29-1.55)	<.0001
Midazolam			
C_max_ (ng/mL)	0.15 (60)	0.02 (36)	
Ratio to control (90% CI)	1	0.15 (0.11-0.21)	<.0001
*t*_½_ (h)	2.63 (36)	1.67 (39)	
Ratio to control (90% CI)	1	0.63 (0.50-0.80)	.0043
*t*_max_ (h)	0.50 (0.25-6.0)	0.50 (0.25-0.5)	.0291
AUC_0–last_ (ng·h·mL^–1^)	0.63 (45)	0.04 (45)	
Ratio to control (90% CI)	1	0.07 (0.05-0.09)	<.0001

Data are presented as geometric means with geometric coefficients of variation (expressed as a percentage), except *t*_max_ for which median and range are shown. Geometric mean ratios between the 2 phases are given with 90% CI. Wilcoxon matched-pairs signed-ranks tests were used to compare *t*_max_ between rifampin and placebo phases. Other pharmacokinetic variables were compared using paired *t* tests on log-transformed data.

Abbreviations: AUC_0–last_, area under the concentration–time curve; AUC_m_/AUC_p_, ratio between metabolite AUC and parent drug AUC; *C*_max_, maximum plasma concentration; CI, confidence interval; Cl, plasma clearance; HM3G, hydromorphone-3-glucuronide; *t*_½_, elimination half-life; *t*_max_, time to reach maximum concentration; *V*_ss_, steady-state volume of distribution.

The concentrations of oral midazolam were markedly reduced by rifampin pretreatment. Rifampin reduced the AUC_0–last_ of midazolam by 93% (ratio to control: 0.07, 90% CI, 0.05-0.09) and the *C*_max_ by 85% (ratio to control: 0.15, 90% CI, 0.11-0.21). Although the median *t*_max_ of midazolam was 0.5 hours in the placebo and rifampin groups, the range of *t*_max_ in the rifampin group was significantly lower (range 0.25–0.5 hours) compared with placebo (range 0.25–6 hours) (*P* = .0291). The reduction in individual hydromorphone AUC_0–last_ of each participant is shown in Supplemental Digital Content 2, Figure 2B, http://links.lww.com/AA/D257.

### Effect of Rifampin on Pharmacodynamic Parameters After Oral and Intravenous Hydromorphone

**Table 3.** Pharmacodynamic Effects of Oral and Intravenous Hydromorphone After Placebo or Rifampin

Parameters	Placebo Phase (Control)	Rifampin Phase	*P*
Intravenous hydromorphone (0.02 mg/kg)			
Drug effect (VAS, mm × hour)	80.25 (13.0-253.75)	87.50 (24.75-298.75)	.8139
Reduction in pupil diameter (mm × hour)	10.59 (3.88-19.23)	7.03 (3.60-17.83)	.0652
Cold pressor threshold (seconds × hour)	77.00 (41.50-160.75)	31.88 (–46.50 to 179.75)	.1575
Oral hydromorphone (2.6 mg)			
Drug effect (VAS, mm × hour)	21.13 (0.00-151.75)	18.13 (0.00-148.25)	.8740
Reduction in pupil diameter (mm × hour)	1.09 (0.93-2.28)	0.74 (1.78-2.45)	.3465
Cold pressor threshold (seconds × hour)	35.25 (–63.25 to 128.75)	9.13 (–139.00 to 90.75)	.1952

Data are presented as median areas under effect–time curve (AUC_0–6_) with ranges. Wilcoxon matched-pairs signed-ranks tests were used to compare time-effect curves between rifampin and placebo phases. Pharmacodynamic effects were studied for 6 h after administration of hydromorphone

Abbreviation: VAS, visual analog scale.

**Figure 3.**
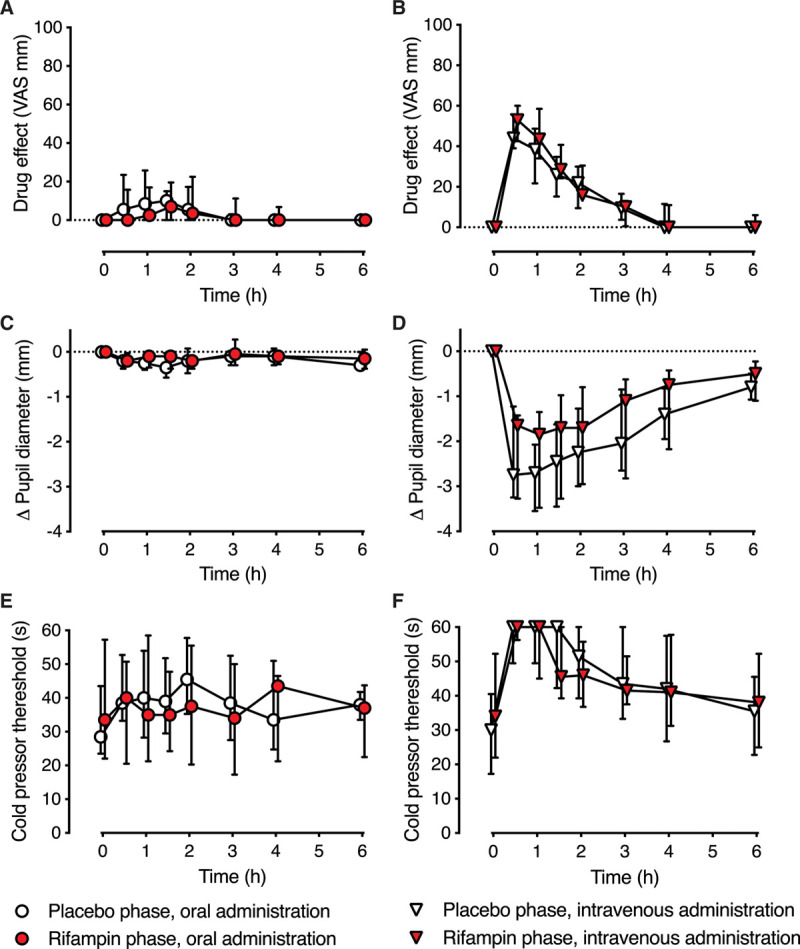
Effect of rifampin pretreatment on the pharmacodynamic effects of hydromorphone. The pharmacodynamic parameters are shown for the oral phase on the left and for the intravenous phase on the right. Changes in the (A, B) self-reported drug effect (VAS), (C, D) change (ie, reduction) in pupil diameter (mm), and (E, F) pain threshold in the cold pressor test(s) are shown after oral (2.6 mg) and intravenous (0.02 mg/kg) hydromorphone in 12 healthy volunteers. Data are presented as medians with interquartile ranges. Data points have been slightly nudged on the x-axis to enhance readability. VAS indicates visual analog scale.

Rifampin did not significantly influence any pharmacodynamic parameters after oral or intravenous hydromorphone (Figure 3; Table 3). The median AUC of perceived hydromorphone effect on the visual analog scale was similar after oral hydromorphone in the rifampin and placebo phases and after intravenous hydromorphone. Reduction in the AUC_0–6_ of pupil diameter tended to be smaller after intravenous hydromorphone in the rifampin phase (median of 7.0 mm × hour) compared with placebo (median of 10.6 mm × hour), although this difference was not significant (*P* = .0652). After oral hydromorphone, the median reduction in pupil diameter was small in the placebo and rifampin phases. Median AUCs of cold pressor threshold were lower, albeit not statistically significant, in the rifampin phase compared with placebo after intravenous hydromorphone (31.9 vs 77.0 seconds × hour, *P* = .1575) and after oral hydromorphone (9.1 vs 35.3 seconds × hour, *P* = .1952). In general, variation in the pharmacodynamic responses of the participants was high.

## DISCUSSION

In healthy volunteers, rifampin reduced the areas under the plasma concentration–time curves of oral and intravenous hydromorphone by 43% and 26%, respectively. Further, the maximum plasma concentration of oral hydromorphone was reduced by 37%, and *F* was reduced from 33% to 26% in the rifampin phase compared with placebo. Rifampin increased the metabolism of hydromorphone to HM3G, as the HM3G-to-hydromorphone metabolic ratio increased by 50% and 42% after oral and intravenous administration, respectively.

The reduction of hydromorphone concentrations by rifampin was clear, particularly in the oral phase. This decreased bioavailability of hydromorphone was most likely due to an increase in its presystemic metabolism. In relative terms, the reduction in the bioavailability of hydromorphone was equal to a 22% reduction in AUC, while the increase in clearance was equal to a 26% reduction in AUC, indicating that induction of presystemic and systemic metabolism were roughly equally involved. Of note, the HM3G-to-hydromorphone AUC ratio was 3 times higher after oral compared with intravenous hydromorphone, consistent with the formation of the HM3G metabolite already during presystemic metabolism. This metabolic ratio was roughly similarly increased by rifampin after oral and intravenous hydromorphone, suggesting that the presystemic metabolism occurs primarily in the liver and not in the intestinal wall.

Rifampin is an antimicrobial drug used worldwide to treat severe bacterial infections. These conditions are often associated with severe pain. Rifampin is also a prototypical CYP enzyme inducer drug that is often used as an index inducer in clinical drug–drug interaction studies.^4,24^ Other strong CYP inducers include carbamazepine and phenytoin that are mainly used to manage epilepsy. Enzyme induction using rifampin as a model drug has been shown to severely decrease the concentrations of opioids that are CYP enzyme substrates. Pretreatment with rifampin decreased the AUCs of oxycodone by 86%,^10^ buprenorphine by 70%,^9^ and tramadol active M1 metabolite by 58%^12^ after oral administration. In addition, rifampin treatment reduced the analgesic effect of transdermal fentanyl^7,8^ and induced withdrawal symptoms in patients using methadone.^6,11^ In contrast to these opioids, morphine has very limited CYP metabolism, as it is mainly metabolized to glucuronides by UGT2B7. Nevertheless, rifampin was reported to reduce the AUC of oral morphine by 28% and *C*_max_ by 41%.^20^ In light of the present study, the magnitude of the interaction between rifampin and hydromorphone is similar to the previously reported interaction with morphine.^20^ Further, the induction of hydromorphone metabolism by rifampin relatively increases the exposure to HM3G, potentially increasing the risk for neuroexcitatory adverse effects, such as opioid tolerance and hyperalgesia.^17,18^ Taken together, appropriate doses for morphine and hydromorphone have to be carefully selected when patients are cotreated with enzyme inducers. However, considering that the reductions in hydromorphone concentrations were moderate, we believe that adjusting the dose in a clinical situation should be feasible.

There may be several mechanistic causes for the pharmacokinetic interaction between rifampin and hydromorphone. First, a plausible explanation is the induction of UGT2B7, the main enzyme that metabolizes hydromorphone, by rifampin. Several in vitro studies have reported induction of UGT enzymes by rifampin.^25–28^ However, whether rifampin induces UGT enzymes in a clinical setting remains poorly characterized. In 1 study, rifampin slightly decreased the concentrations of drugs that are eliminated mainly via glucuronidation.^29^ In our study, the glucuronide-parent drug metabolic ratio was increased by 50%, indicating that UGT induction can account for a major part of the observed interaction. A possible secondary explanation could be the induction of P-glycoprotein, a drug efflux protein for which hydromorphone has been suggested to be a substrate in vitro.^30^ Rifampin is a known inducer of P-glycoprotein in the intestinal wall and the kidney,^31^ and induction of P-glycoprotein–mediated efflux of morphine and morphine-6-glucuronide into the biliary tract or the gut lumen was speculated to explain the morphine-rifampin interaction, at least in part.^20^ However, as the lipophilicity of hydromorphone is greater than that of morphine,^32^ the effect of P-glycoprotein on the efflux of hydromorphone may be of less importance. Third, similar to morphine, induction of minor CYP-mediated metabolism routes may have a role in the increased clearance of hydromorphone.^20^ Detailed mechanistic studies will be needed to fully understand the interaction.

The pharmacodynamic effects of hydromorphone were not statistically significantly changed by rifampin despite the decreased exposure to hydromorphone in the rifampin phase. We, nevertheless, believe that pharmacokinetic interaction is clinically relevant. Due to large variation in the pharmacodynamic measurements, our study was not sufficiently powered to detect significant differences between rifampin treatment and placebo. On the other hand, pupillometry is a rather sensitive tool to assess the pharmacodynamic effects of opioids and also to estimate their plasma concentrations.^33^ After intravenous hydromorphone, the change in the pupil diameter tended to be smaller in the rifampin phase compared with the placebo phase (Figure 3D), although it did not reach significance at the 5% level (*P* = .0652). Considering the minimal effect of the small, single oral hydromorphone dose on pupil size in the placebo phase, it is not surprising that the effect of rifampin could not be detected. Due to potentially large variation in bioavailability of hydromorphone,^19^ we had to minimize the oral dose of hydromorphone to ensure the safety of the participants. To further characterize the effects of rifampin on the pharmacodynamics of hydromorphone, a larger additional study would be warranted, preferably in patients with a clinically relevant dose of hydromorphone.

The study benefitted from a randomized crossover design that is a statistically efficient method and minimizes confounding factors as every volunteer serves as their own control. The route of hydromorphone administration was not randomized (ie, each volunteer received the oral dose followed by the intravenous dose), which could potentially lead to time-dependent changes in metabolism and affect the comparisons between the intravenous and oral routes. However, our aim was not to compare the routes of administration, but rather to compare rifampin treatment with placebo. The chosen approach minimized the rifampin exposure of volunteers while enabling us to study 2 routes of administration. The participants were blinded to the interventions, although many of them noticed the typical change in color of their urine when they were taking rifampin. We demonstrated that enzyme induction had indeed taken place in all volunteers using a clinically irrelevant dose of midazolam (0.1 mg), an index substrate of CYP3A.^24,34^

In conclusion, we found that pretreatment with the CYP enzyme inducer rifampin significantly decreases systemic exposure to hydromorphone. A larger sample size would have been needed to detect a significant difference in pharmacodynamic responses. The magnitude of the pharmacokinetic interaction between rifampin and hydromorphone is comparable to the previously reported interaction between rifampin and morphine^20^ and needs to be considered when managing pain in patients who are treated with a CYP inducer drug. The dose of particularly oral hydromorphone may need to be adjusted when a patient is treated with an enzyme inducer or when the inducer treatment is withdrawn.

## ACKNOWLEDGMENTS

The authors thank Eija Mäkinen-Pulli, Department of Clinical Pharmacology, HUS Helsinki University Hospital, Helsinki, Finland; Lisbet Partanen, Department of Clinical Pharmacology, HUS Helsinki University Hospital, Helsinki, Finland; Pauliina Simanainen, Department of Anaesthesiology, Intensive Care, and Pain Medicine, HUS Helsinki University Hospital, Helsinki, Finland; and Päivi Vesterinen, Department of Anaesthesiology, Intensive Care, and Pain Medicine, HUS Helsinki University Hospital, Helsinki, Finland, for their skillful assistance.

## DISCLOSURES

**Name:** Terhi J. Lohela, MD, PhD.

**Contribution:** This author helped design the study, conduct the study, analyze the data, and prepare the manuscript.

**Name:** Satu Poikola, MD.

**Contribution:** This author helped conduct the study and prepare the manuscript.

**Name:** Mikko Neuvonen, MSc.

**Contribution:** This author helped design the study, conduct the study, and prepare the manuscript.

**Name:** Mikko Niemi, MD, PhD.

**Contribution:** This author helped design the study and prepare the manuscript.

**Name:** Janne T. Backman, MD, PhD.

**Contribution:** This author helped design the study, conduct the study, analyze the data, and prepare the manuscript.

**Name:** Klaus T. Olkkola, MD, PhD.

**Contribution:** This author helped design the study, conduct the study, analyze the data, and prepare the manuscript.

**Name:** Tuomas O. Lilius, MD, PhD.

**Contribution:** This author helped design the study, conduct the study, analyze the data, and prepare the manuscript.

**This manuscript was handled by:** Ken B. Johnson, MD.

## Supplementary Material


