# Supplementary material for: Rifampin Reduces the Plasma Concentrations of Oral and Intravenous Hydromorphone in Healthy Volunteers
Source: Anesth Analg. 2020 Nov 6;133(2):423–34. doi: 10.1213/ANE.0000000000005229 (PMC8257471; doi:10.1213/ANE.0000000000005229)
Supplement: Supplementary file 2 [file ane-133-423-s002.docx]

**Supplementary Figures**

**Supplementary fig. 1.** Trial design. A two-phase, paired, placebo-controlled, randomized crossover study design at intervals of 6 weeks was used. Twelve participants were given in randomized order either 600 mg oral rifampin or placebo once daily at 8 PM for 8 days (black arrows). Hydromorphone (orange arrows) was administered orally (p.o.) on day 6 and intravenously (i.v.) on day 8 of a phase.

**Supplementary fig. 2.** Effect of rifampin on the individual areas under the time-concentration curves of hydromorphone (AUC0–last) after oral administration (2.6 mg, A) and intravenous administration (0.02 mg/kg, B). Individual data points are presented for each volunteer.
